# GERD outcome after bariatric surgery: A protocol for systematic review and meta analysis

**DOI:** 10.1097/MD.0000000000019823

**Published:** 2020-05-08

**Authors:** Abdel-Naser Elzouki, Muhammad-Aamir Waheed, Salah Suwileh, Dayyan M. Adoor, Osama Tashani, Abdul-Badi Abou Samra

**Affiliations:** a Hamad General Hospital, Hamad Medical Corporation; b Weill Cornell Medicine; c College of Medicine, Qatar University, Qatar; d School of Clinical and Applied Sciences, Leeds Beckett University, Leeds, United Kingdom.

**Keywords:** bariatric surgery, gastroesophageal reflux, laparoscopic sleeve gastrectomy, meta-analysis, Roux-en-Y gastric bypass, systematic review

## Abstract

**Background/Aim:**

Obesity is associated with increased incidence of gastroesophageal reflux disease (GERD), and it has been suggested that GERD symptoms may be improved by weight reduction. However, various patterns of bariatric surgery may affect symptoms of GERD due to the changed anatomy of stomach and esophagus. The aim of this systematic review and meta-analysis is to analyze the effect of bariatric surgery on GERD.

**Materials and methods:**

A systematic literature search was performed using PubMed, EMBASE, and the Cochrane Library from January 2005 to January 2019, combining the words obesity, gastroesophageal reflux with different types of bariatric surgery and weight loss. The methodological quality of randomized controlled trials and non-randomized controlled trials published in English and have at least 1-year follow-up data were included and assessed by Cochrane Collaboration's tool for assessing risk bias and Newcastle–Ottawa scale. Only clinical trials were included, and case series or case reports were excluded.

**Results:**

We anticipate that our review will provide the exact estimates of the burden and phenotype of GERD among patients that have undergone bariatric surgery.

**Conclusion:**

GERD may improve in obese patients who underwent laparoscopic sleeve gastrectomy (LSG); however, the most favorable effect is likely to be found after Roux-en-Y gastric bypass surgery.

**Prospero registration number:**

CRD42018090074.

## 1 Introduction and rationale

Obesity is a major concern all over the world affecting mainly adult and adolescent population.^[1]^ It is associated with a high incidence of many preventable co-morbidities including diabetes, cardiovascular disease, and cancer.^[2]^ For most patients, bariatric surgery remains the only option for the treatment of obesity when dietary interventions and medications fail. Currently used bariatric surgery [laparoscopic sleeve gastrectomy (LSG) and laparoscopic Roux-en-Y gastric bypass (LRYGB)] lead to various degree of weight loss.^[3]^ However, there has been a rising incidence in complications associated with these procedures. Gastroesophageal reflex disease (GERD) in particular has been the subject of such concerns.^[4]^ The prevalence of clinically relevant GERD associated with bariatric surgery is variable but has been reported as ranging between 7% and 14%.^[5–7]^ Uncertainty however remains regarding the exact prevalence as well as the case burden especially among patients with different clinical phenotypes such as metabolic syndrome and obesity multi-morbidities. Factors accounting for this apparent lack of agreement on the data includes differences in background characteristics of patient populations as well as the clinical phenotypes of the patient population studied. In this synthesis, we intend to carry out a systematic review and meta-analysis of current evidence with the view to clarify the exact burden of GERD among patients that have undergone bariatric surgery.

## 2 Objective

The key objective of this study is to carry out a systematic review with meta analysis of controlled trials with the view to evaluate and clarify the exact burden of GERD following main modalities of bariatric surgery procedures (LSG and LRYGB).

## 3 Overview

This study will be conducted in line with the recommendation of Preferred Reporting Items for Systematic Reviews and Meta-Analyses guidelines^[8]^ for meta-analyses of healthcare interventions. Additionally, the current protocol outline adheres strictly to the Preferred Reporting Items for Systematic Reviews and Meta-Analyses Protocols (PRISMA-P).^[9]^ This protocol is registered in International Prospective Register of Systematic Reviews (registration number: CRD42018090074).

## 4 Methodology

### 4.1 Population

This review study population will comprise of studies reporting on frequency of GERD following bariatric surgery (LSG and LRYGB).

### 4.2 Intervention

Bariatric surgery (LSG and LRYGB).

#### 4.2.1 Study outcomes

Development of newly onset, worsened, or improved GERD among this population.

### 4.3 Study eligibility criteria

#### 4.3.1 Inclusion criteria

In this review, we will include studies that meet the following inclusion criteria:

Age > 18 years.
History of bariatric surgery.
All indications for bariatric surgery.
Studies reporting on outcomes that includes GERD and its prevalence.
Studies published in English language published from January 1, 2005 to January 31, 2019.

### 4.4 Exclusion criteria

All other studies that fail to satisfy the afore-mentioned inclusion criteria.

### 4.5 Literature sources

Relevant studies for this review will be sources from the following publicly available databases

- Controlled clinical trials
- Cochrane Controlled Trials Register (CCTR)
- Cochrane Database of Systematic Reviews (CDSR)
- EMBASE
- Database of Abstracts of Reviews of Effectiveness (DARE)
- MEDLINE
- Science Citation Index (SCI)
- Index to Scientific and Technical Proceedings (ISTP)
- National Research Register (NRR)

### 4.6 Search strategy

This study's search strategy will aim to identify published controlled clinical trials between January 1, 2005 and January 1, 2019 using the following medical search operators gastroesophageal reflux[tiab] OR bariatric surgery[tiab] OR laparoscopic sleeve gastrectomy[tiab] OR laparoscopic Roux-en-Y gastric bypass[tiab] AND (Clinical Trial[ptyp] AND “humans”[MeSH Terms]) from the following sources:

- Controlled clinical trials
- Cochrane Controlled Trials Register (CCTR)
- Cochrane Database of Systematic Reviews (CDSR)
- EMBASE
- Database of Abstracts of Reviews of Effectiveness (DARE)
- MEDLINE
- Science Citation Index (SCI)
- Index to Scientific and Technical Proceedings (ISTP)
- National Research Register (NRR)

### 4.7 Criteria for study selection

All studies that met eligibility criteria will be abstracted from publicly available electronic databases and imported in study specific database. Duplicates will be removed. Two reviewers will each independently assess full text of for inclusion in the review.

## 5 Interventions

Studies included this review will be those that report on patients that have undergone bariatric surgery (LSG and LRYGB) for a wide variety of reasons and reported on the prevalence of GERD in those populations.

### 5.1 Data collection/abstraction

Data will be collected in a study specific case record form which was initially trialed on a randomly selected study. This data will then be transferred to a Microsoft specific database by the first author's last name and the year of publication.

### 5.2 Study bias risk assessment

We will carry out risk of bias assessment of the all included studies and ascertain the magnitude of their bias disposition on the review outcome estimate.

### 5.3 Data analyses/synthesis

#### 5.3.1 Estimation/calculation of effect sizes

We will determine the pooled estimates of our review outcome from effect sizes [relative risk (RR), odds ratio (OR), and count data] of the constituent studies by random effect models. We will generate other models (fixed effects and inverse heterogeneity models) for comparison.

Secondary outcomes will be calculated using the same procedure as for primary outcome.

### 5.4 Analytical software for data synthesis

All statistical analysis will be carried out with MetaXL software (version 5.3 © EpiGear International Pty Ltd ABN 51,134,897,411 Sunrise Beach, Queensland, Australia, 2011–2016).

## 6 Statistical analysis

### 6.1 Strategy for dealing with missing data

In the event of missing data from any of the reviews outcome of interest, we will request this from the corresponding authors of the studies concerned.

### 6.2 Sensitivity analysis

We will carry out sensitivity analysis where necessary to ascertain the exact effect of individual studies on the overall review outcome.

### 6.3 Ascertainment of heterogeneity

We will determine heterogeneity by estimating and comparing Cochran Q and *I*^2^ statistics.

## 7 Discussion

We anticipate the outcome of this review will provide clarity regarding the exact phenotype and burden of GERD following bariatric surgery. This is of utmost importance both for clinical commissioning of measures to address this but also for laying foundation for further studies.

## Author contributions

**Conceptualization:** Abdel-Naser Elzouki, Muhammad-Aamir Waheed.

**Data curation:** Abdel-Naser Elzouki, Muhammad-Aamir Waheed, Salah Suwileh, Dayyan M. Adoor, Osama Tashani.

**Formal analysis:** Osama Tashani, Abdel-Naser Elzouki.

**Investigation:** Abdel-Naser Elzouki.

**Methodology:** Abdel-Naser Elzouki, Muhammad-Aamir Waheed, Salah Suwileh, Dayyan M. Adoor, Osama Tashani.

**Project administration:** Abdel-Naser Elzouki, Muhammad-Aamir Waheed, Abdul-Badi Abou Samra.

**Resources:** Abdel-Naser Elzouki, Muhammad-Aamir Waheed, Abdul-Badi Abou Samra.

**Validation:** Salah Suwileh, Dayyan M. Adoor.

**Writing – original draft:** Abdel-Naser Elzouki, Muhammad-Aamir Waheed.

**Writing – review & editing:** Abdel-Naser Elzouki, Abdul-Badi Abou Samra, Osama Tashani.
